# Mind Cure

**Published:** 1888-06

**Authors:** 


HALL'S
JOURNAL OF HEALTH

TRUTH DEMANDS NO SACRIFICE; ERROR CAN MAKE NONE.

VOL. 35 JUNE, 1888. No. 6.

### MIND CURE.

It will have been observed by our readers that as previously announced we have made arrangements with Mrs. Dr. Densmore of New York City, to give in the colums of the Journal, a comprehensive statement of the Mind Cure as taught and practiced by the pioneers of this latest absurdity including Mrs. Eddy and such of her pupils as have opened separate schools at "cut rates."

The first of this series of articles appeared in our May issue being substantially the same as formerly appeared in a publication entitled "Earnest Words," which has been suspended, and it is proposed to continue the subject in succeeding numbers of the Journal, until the complete system as taught by the enthusiasts of the new school, for tuition fees ranging from $25 to $300, shall have been made clear to our patrons in all its verbose transcendentalism.

The basic theory of these heralds of reform is that there is no such thing as sickness, sin and death; that matter itself has no real existence; that *mind*, being of the spirit spiritual, is the all in all of human existence; all contrary views are the results of a diseased and distorted imagination, which to eradicate is the office of the Mind Cure School.

One who has been accustomed to different views will be slow to admit that he has been all his life mistaken in supposing that his body was something more than a delusion; that the superb mansion across the way is not altogether the product of a mental effort

"Begot of nothing but vain fantasy,"

charmed into imaginary being by a wish; and such a difference too in wishes: Let the reader imagine himself lying helpless upon a sick-bed, with a broken leg or a cancer preying upon some vital part. The Mind Cure practitioner enters the room, to effect a cure by a process of mental subordination. You think you see her, but that is a delusion. She does not examine your person for that is a myth, she does not feel your pulse nor examine into your ailments, for there is no pulse, no ailment; it is all a misconception, a phantasm. She appears to seat herself at the bedside—but this too is a delusion, there is no room, no bed, no ailment; she begins to operate upon the fancied disease—you are not sick; you only think you're sick, your leg is not broken; in the light of absolute truth you have no leg to break—no body to mend, it is all a delusion—that which is commonly called a leg is only "the reflection of a reflection," think of yourself as well and your imaginary disease will disappear.

We are told of extraordinary instances of Mind Cure. We are also informed of some lamentable failures, but the blame is not laid to the operators, by the new school, but rather to the patients whose mind is unreceptive to the transmission of intangible doses of mental physic.

We could understand Dr. Evans when he affirmed in his Divine Law of Cure, that all forms of matter are the phenomena of spirit. The inventor, the architect and the builder, work by a preconceived plan; an idea; a mental picture, giving it form and expression in matter; but when it is affirmed by the Eddyite philosophers that matter itself has no existence, that the body is unreal and disease a misconception, we confess to a stupid unbelief in the new school and wonder that it has gained so many converts.

